# Perceived social support as a mediator between left-behind experience and subjective well-being in Chinese college students

**DOI:** 10.7717/peerj.20567

**Published:** 2026-01-09

**Authors:** Biwei Zhou, Lei Zhang

**Affiliations:** School of Mental Health, Bengbu Medical University, Bengbu, Anhui, China

**Keywords:** Left-behind experience, Perceived social support, Subjective well-being, College students, Mediation

## Abstract

**Background:**

Left-behind experience (LBE), where children are separated from one or both parents due to parental migration for work, has been shown to negatively affect college students’ psychological well-being. This study explores the relationship between LBE and subjective well-being (SWB) among college students and investigates whether perceived social support (PSS) mediates this relationship.

**Methods:**

A cross-sectional survey was conducted with 1,228 undergraduate students across four Chinese universities. Participants completed the Perceived Social Support Scale (PSSS) and the Subjective Well-Being Scale (SWB). Statistical analyses, including independent t-tests, Pearson correlations, and mediation analysis (using SPSS PROCESS), were performed to test the hypotheses.

**Results:**

Students with LBE reported lower levels of both PSS and SWB compared to their non-LBE peers. PSS was found to fully mediate the relationship between LBE and SWB. Specifically, LBE negatively affected PSS, which in turn positively influenced SWB. The direct effect of LBE on SWB was non-significant, highlighting the importance of PSS in this relationship.

**Conclusions:**

The study emphasizes that LBE itself does not directly dictate developmental outcomes. Instead, it influences college students through the reduction of perceived social support. These findings suggest that enhancing social support systems in universities and communities can significantly improve the well-being of college students with LBE, and this approach has potential implications beyond the Chinese context.

## Introduction

Since the 1990s, China’s rapid and robust economic growth has led to significant rural-to-urban migration, as many rural laborers have moved to cities in search of higher wages. This migration trend has resulted in the emergence of a unique demographic: “left-behind children” in rural areas. According to the China Children’s Development Report, by 2024, it is projected that over 40 million children in rural China will be left behind, with 96% of them being cared for by their grandparents, while 4% are under the guardianship of other relatives or friends (China Development Research Foundation, 2024). Research indicates that most left-behind children face significant psychological burdens, lack a sense of security, and long for the opportunity to live with their parents (Wu & Li, 2015). Due to prolonged parental absence, they often exhibit weaker parent-child attachment and lower life satisfaction than their non-left-behind peers (Li & Liu, 2013; Cai, Tan & Zhao, 2025). As time has passed, some of these early left-behind children have entered adulthood, with a number successfully gaining admission to universities through their efforts. These college students with left-behind experience (LBE) represent the continuation of the left-behind phenomenon into the next generation. This raises an important question: How does the experience of being a left-behind child during childhood affect individual development in adulthood?

An increasing number of studies have begun to examine the effects of LBEs on various aspects of college students’ lives, including their emotional well-being, behavioral tendencies, and social adaptation. LBE is typically defined as a situation where children are separated from one or both parents who have migrated for work for an extended period (usually exceeding 1 year) (Yang, Feng & Cui, 2014). Substantial evidence indicates that LBEs constitute an adverse childhood experience with detrimental consequences for mental health (Shen & Zhang, 2018; Ye et al., 2020). Students with a left-behind history report significantly higher levels of depression and anxiety, coupled with lower scores in subjective well-being (SWB), compared to their peers without such experiences (Chen, 2025; Wang, 2008). Subjective well-being (SWB) refers to an individual’s comprehensive assessment of their emotional perception of life, reflecting their level of psychological development and positive psychological functioning, including feelings of energy, life satisfaction, emotional health, and behavioral control (Diener, 1984). This concept reflects both the health status of psychological development and the level of positive mental states experienced by individuals. The findings consistently indicate that these experiences, recognized as a form of complex trauma, exert a detrimental influence on the SWB of college students (Zhang & Xu, 2020; Wen & Zeng, 2012; Li et al., 2009; Liu & Wang, 2017). For instance, Wang (2010) found that college students with LBEs exhibited poor SWB, characterized by a dominance of negative emotions. Corroborating this, Zhou et al. (2014) demonstrated that LBE serves as a significant negative predictor of SWB. These findings collectively underscore that the effects of parental separation in childhood are enduring, exerting a long-term negative impact on subjective well-being.

**Figure 1:**
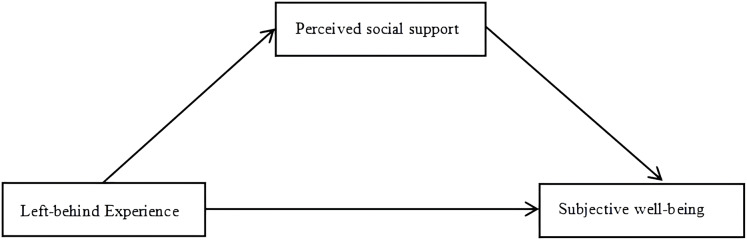
Hypothetical model.

Perceived social support (PSS) is understood as the emotional satisfaction derived from the perception of being respected, supported, and understood, with an emphasis on an individual’s self-perception and emotional experience of the support received (He, 2013). For individuals with LBEs, research indicates that a longer duration of parental absence is associated with diminished PSS (Wang et al., 2022), demonstrating how this adverse childhood event can negatively impact support perception into adulthood (Ma et al., 2022). According to buffering theory, PSS can help alleviate or counteract the negative effects of adverse events and plays a vital role as a mediator between psychological stress and health (Luo & Mu, 2017). Research indicates that social support is a significant positive predictor of subjective well-being. Higher levels of perceived social support among college students are strongly associated with increased levels of subjective well-being, suggesting that individuals with strong social connections tend to report greater life satisfaction and overall mental well-being (Matsuda et al., 2014). Perceived social support is a direct contributor to the positive emotional experiences of college students. For college students with experiences of parental absence, improving the social support environment can assist them in overcoming the adverse effects caused by the challenges of growing up in such conditions (Luo & Zhou, 2017). Similar phenomena have been observed internationally. For example, in Latin American and Eastern European countries, children left behind due to parental migration also experience significant emotional and psychological challenges. These studies highlight the global relevance of the Left-behind experience and its impact on adolescent mental health and subjective well-being (DeWaard, Nobles & Donato, 2018). Building on the previous analysis, this study explores how Left-behind experiences affect the subjective well-being of college students in China. We hypothesize that LBEs serve as a significant negative predictor of subjective well-being among college students. Additionally, we propose that perceived social support plays a critical mediating role in the relationship between LBEs and subjective well-being. Specifically, LBEs exert an indirect influence on college students’ subjective well-being through perceived social support (Fig. 1).

## Methods

### Participants

This study employed a multistage cluster sampling method with stratification and random selection. In the first stage, purposive sampling was used to select universities from Hubei and Anhui provinces in China. The second stage involved stratified sampling, selecting four universities from each province: two comprehensive universities and two universities focused on science and engineering. In the third stage, a combination of stratified and cluster sampling was applied to randomly select 2–3 classes per university, representing all academic years from freshman to senior. All students in the selected classes were invited to complete a questionnaire. To ensure sample validity, invalid responses, including incomplete or extreme patterns of answers, were excluded after data collection. After this filtering process, 1,228 valid responses were obtained, resulting in an effective response rate of 94.5%. Among the valid responses, 540 were from male students (44%), and 688 were from female students (56%). The distribution across academic years was as follows: 342 freshmen (27.9%), 310 sophomores (25.2%), 228 juniors (18.6%), and 348 seniors (28.3%). Additionally, 398 students (32.4%) were from urban areas, and 830 (67.6%) were from rural areas.

### Materials

#### Left-behind experience questionnaire

According to existing studies, we define “Left-behind experience” as the situation in which one or both parents migrate for work to other regions during the individual’s growth period (0–17 years), resulting in the individual being separated from their parents and remaining at home for more than 1 year (Yang, Feng & Cui, 2014). Respondents who answer “Yes” are classified as having “Left-behind experience,” while those who answer “No” are classified as having “Non-Left-behind experience”.

#### Perceived Social Support Scale

The perceived level of social support in this study was assessed using the Chinese version of the Perceived Social Support Scale (PSSS), which is based on the 1988 study by Zimet (Heitzmann & Kaplan, 1988; Wang, Wang & Ma, 1999). The scale consists of 12 items, mainly assessing the level of social support that individuals perceive, which encompasses three dimensions: support from family, support from friends, and support from others. A 7-point Likert scale was utilized, where 1 represents strong disagreement and 7 indicates strong agreement. A higher overall score suggests a greater perception of social support. In this study, the Cronbach’s α coefficient of the scale was 0.94.

#### Subjective Well-Being Scale

The Subjective Well-Being Scale (SWB) is a measurement tool developed by the National Center for Health Statistics in the United States to assess individuals’ statements regarding their well-being, consisting of 33 items. In 1996, Duan (1996) revised the Chinese version of the SWB, retaining the first 18 items. The scale primarily assesses individuals’ subjective feelings and experiences regarding energy, life satisfaction, emotional well-being, and behavior control over the past month. Higher scores on the scale reflect a higher level of subjective well-being. In this study, the Cronbach’s α coefficient of the scale was 0.85.

### Data collection and statistical analyses

All data were collected online using the Wenjuanxing app from March 20 to 25, 2024, with participants instructed to provide truthful responses and complete the surveys anonymously. Before the survey, approval was obtained from the student administrator, and verbal informed consent was acquired from the participants. The study received approval from the Ethics Committee of Bengbu Medical University (Approval No.: 2023-256).

Data analysis was performed using SPSS version 24.0 statistical software. An independent-sample t-test was employed to examine the differences in the scores across various variables between students with and without Left-behind experience. Independent-sample t-tests and one-way ANOVA were conducted to examine the gender- and grade-related differences in the scores across various variables for students with Left-behind experience. Pearson correlation analysis was utilized to investigate the relationships between the variables. Mediation effects were tested using the Process plugin and bias-corrected percentile bootstrap method.

## Result

### Common method bias test

All data in this study were collected using self-report methods, which may introduce common method bias that could influence the results. The Harman single-factor test was employed to examine the presence of common method bias. The test results revealed that three factors had eigenvalues greater than 1, and the variance explained by the first factor was 38.1%, which is below the critical threshold of 40%. This suggests that there is no significant common method bias in the data of this study.

### Comparison of scores of various scales among college students with or without LBE

Independent sample t-tests revealed that college students with LBEs scored significantly lower across all scale totals and sub-dimensions compared to their counterparts without such experiences. Further analysis of gender- and grade-related differences in various variables for students with LBEs indicated that male students reported significantly higher levels of family support than their female counterparts. Furthermore, significant grade-level differences in peer support were observed, with *post-hoc* analyses indicating that first-year students reported significantly higher peer support compared to students in higher years (Table 1).

**Table 1:** Comparison of scale scores (M ± SD) between groups with and without LBE.

Variables	LB (*n* = 588)	NLB (*n* = 640)		College students with LBE							
			t	Male (*n* = 251)	Female (*n* = 337)	t	Freshmen (*n* = 146)	Sophomore (*n* = 149)	Junior (*n* = 112)	Senior (*n* = 181)	f
PSS	5.00 ± 1.05	5.39 ± 1.04	6.56***	5.04 ± 1.06	4.97 ± 1.04	7.84	5.16 ± 0.95	4.87 ± 1.07	4.89 ± 1.05	5.05 ± 1.09	2.46
Family support	5.01 ± 1.27	5.48 ± 1.15	6.74***	5.18 ± 1.21	4.89 ± 1.29	2.77*	5.13 ± 1.15	4.89 ± 1.39	4.92 ± 1.19	5.08 ± 1.30	1.23
Friend support	5.19 ± 1.08	5.46 ± 1.08	4.30***	5.18 ± 1.11	5.19 ± 1.06	0.12	5.41 ± 0.98	5.09 ± 1.04	5.02 ± 1.11	5.20 ± 1.14	3.36*
Other support	4.79 ± 1.19	5.24 ± 1.19	6.49***	4.76 ± 1.21	4.83 ± 1.19	0.75	4.95 ± 1.12	4.64 ± 1.12	4.73 ± 1.19	4.86 ± 1.24	1.94
SWB	4.22 ± 0.71	4.37 ± 0.74	3.44***	4.18 ± 0.73	4.25 ± 0.70	1.11	4.34 ± 0.79	4.28 ± 0.70	4.13 ± 0.64	4.13 ± 0.68	3.41

**Notes:**

* *P* < 0.05.

*** *P* < 0.001.

LB, left-behind, NLB, non-left-behind.

### Correlational analysis between variables

As the “Left-behind experience” is a categorical variable, it was dummy-coded (0 for Non-Left-behind experience, 1 for having Left-behind experience) before conducting the correlational analysis, transforming it into a pseudo-variable. Table 2 reveals a significant negative correlation between perceived social support and subjective well-being with Left-behind experience. Additionally, perceived social support and subjective well-being show a significant positive correlation.

**Table 2:** Correlation analysis between variables (r).

Variables	M	SD	LBE	PSS
LBE	0.48	0.49	1	
PSS	5.21	1.06	−0.19**	
SWB	4.31	0.73	−0.10**	0.46**

**Note:**

** *P* < 0.01.

### Examination of the mediating effect of perceived social support

**Figure 2:**
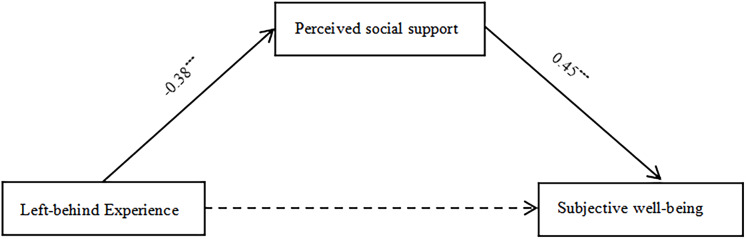
Test of mediating effect of perceived social support.

The mediation effect of perceived social support between LBEs and college students’ subjective well-being was examined using Model 4 of the PROCESS macro in SPSS, controlling for gender, grade, and hometown, as shown in Table 3 and Fig. 2. The data indicate that LBEs significantly negatively predicted subjective well-being (β = −0.18, *P* < 0.001), but after including perceived social support as a mediator, this negative relationship was no longer significant (β = −0.01, *P* > 0.001). LBEs significantly negatively predicted perceived social support (β = −0.38, *P* < 0.001), and perceived social support significantly positively predicted subjective well-being (β = 0.45, *P* < 0.001). This suggests that LBEs can only indirectly predict subjective well-being through perceived social support, which fully mediates the relationship between LBEs and college students’ subjective well-being.

**Table 3:** Mediation effect analysis (*n* = 1,228).

Regression equation		Overall fit index			Significance of regression coefficients	
Outcome variables	Predictor variables	R	R^2^	F	β	t
SWB	Gender	0.16	0.03	8.46***	0.10	1.79
	Grade				−0.11	−4.38***
	Place of origin				0.01	0.13
	LBE				−0.18	−3.05**
PSS	Gender	0.19	0.03	10.90***	−0.01	−0.23
	Grade				−0.01	−0.26
	Place of origin				0.05	0.76
	LBE				−0.38	−6.36***
SWB	Gender	0.48	0.23	71.32***	0.11	2.13*
	Grade				−0.10	−4.70***
	Place of origin				0.01	0.24
	PSS				0.45	17.72***
	LBE				−0.01	−0.19

**Notes:**

* *P* < 0.05.

** *P* < 0.01.

*** *P* < 0.001.

Furthermore, the direct impact of the LBE on subjective well-being is −0.010, with the bootstrap 95% confidence interval spanning from [−0.118 to 0.098], which encompasses 0. In contrast, the mediating effect of perceived social support is −0.174, with the Bootstrap 95% confidence interval ranging from [−0.235 to −0.119], which does not include 0 (see Table 4).

**Table 4:** Total, direct, and indirect effects.

Effects	Effect size	BootSE	LLCI	ULCI
Total effects	−0.184	0.061	−0.303	−0.066
Direct effects	−0.010	0.055	−0.118	0.098
Indirect effects	−0.174	0.029	−0.235	−0.119

## Discussion

In recent years, as China’s higher education system has expanded, college students who have experienced left-behind childhoods have become an increasingly significant demographic, and their psychological well-being has garnered growing attention from researchers. Our study indicates that college students with LBEs report significantly lower levels of perceived social support and subjective well-being compared to their peers without such experiences. These findings align with the results of previous studies (Yang, Feng & Cui, 2014; Wang, 2008; Zhou et al., 2014). It is widely believed that students who experienced left-behind childhoods were deprived of parental companionship during crucial developmental years. The absence of their parents has hindered opportunities for establishing intimate communication and emotional bonds. Consequently, this experience often leads to a reduced sense of social support, which, in turn, negatively impacts life satisfaction and contributes to a decline in subjective well-being (Su et al., 2017). Our research also reveals that male students who have experienced being left behind report significantly higher levels of family support compared to their female counterparts. This phenomenon may be attributed to the entrenched “son-preference” mentality prevalent in rural areas of China, where families tend to offer more support and assistance to male children. Regarding academic year, first-year students report significantly higher levels of peer support compared to students in higher years. As freshmen, they are navigating a new environment, often far from home, and unlike their high school experience, they spend less time with teachers. During this transitional phase, their primary source of support comes from classmates and friends, which helps facilitate their adjustment to university life (Luo & Mu, 2017).

Our results reveal a significant positive correlation between perceived social support and subjective well-being. Furthermore, a strong negative correlation was found between perceived social support and LBE, highlighting the profound impact that the LBE has on college students’ social adaptation and mental health (Ning et al., 2024; Lai, 2021). Mediation analysis further shows that perceived social support plays a critical role as a mediator between the LBE and subjective well-being, fully mediating the relationship between these two variables among college students. Students who have experienced being left behind may have spent limited time with their parents, from childhood through university, due to prolonged separations. As a result, they faced challenges independently and dealt with problems without parental support. This prolonged absence has led to a diminished sense of social support.

According to attachment theory, the emotional bond between children and their primary caregivers (typically parents) plays a crucial role in shaping an individual’s later social functioning and mental health (Dignam, 2013). Prolonged separation due to parents working away from home may lead children to develop insecure attachment patterns, which, in turn, can hinder their ability to form social relationships and perceive support in adulthood (Coan, Beckes & Allen, 2013). Additionally, social support buffering theory posits that positive social support can mitigate the psychological effects of stressful or traumatic events (Rubia et al., 2020). Existing research has shown that the LBE reduces perceived social support levels, with low social support serving as a critical mediator in the decline of subjective well-being. Social support is a key determinant of happiness, as the support from others fosters positive emotions. Among the various forms of social support, family support is a strong predictor of college students’ subjective well-being (Xin & Chi, 2001). For college students with a LBE, positive social support acts as a protective factor, promoting both physical and mental well-being and enhancing their subjective well-being (Choi, 2022). The reduction in perceived social support helps explain the negative impact of the Left-behind experience on subjective well-being.

Moreover, when considering the moderating role of perceived social support, the left-behind childhood experience did not show a direct or significant predictive effect on subjective well-being. This suggests that the experience of being left behind during childhood does not directly affect the subjective well-being of college students. Previous studies examining the impact of left-behind childhood experiences on college students have produced inconsistent results. The LBE itself has both positive and negative effects, and its impact on college students varies on an individual level (Li, 2016; Liu et al., 2015). The findings of our study contribute to a deeper understanding of the effects of left-behind childhood experiences, indicating that the experience of being “left behind” lacks sufficient explanatory power to account for individual developmental changes. Furthermore, variations in proximal environmental factors within the left-behind context lead to divergent developmental outcomes. In other words, the left-behind childhood experience does not directly explain individual developmental changes; instead, its effects are mediated through personal and social environmental factors (Xie & Zhang, 2011).

Our study examines the impact of the LBE on the subjective well-being of Chinese college students and explores the mechanisms underlying this effect. The findings emphasize the mediating role of perceived social support and highlight that positive social support can mitigate the negative psychological consequences of the Left-behind experience. This suggests that the LBE itself does not solely determine developmental outcomes; instead, proximal environmental factors play a crucial role, offering a novel perspective on the dynamic relationship between adversity and psychological adaptation. The lack of parental affection during childhood, due to grandparental caregiving, has left a lasting impact. The experiences of parental absence and the left-behind status are largely irreversible, presenting a significant challenge that must be addressed by schools, families, and society. Family support plays a vital role in the subjective well-being of college students. Furthermore, as the primary setting for students’ academic and social lives, universities should provide essential support and care. Our research suggests that universities and relevant departments should prioritize developing social support systems for students with LBEs, particularly by strengthening family interactions, fostering peer support, and integrating campus resources to enhance their subjective well-being. However, our study has several limitations. First, the cross-sectional design restricts the ability to make causal inferences between the variables, underscoring the need for longitudinal studies in the future to validate the underlying mechanisms. Additionally, the heterogeneity of the Left-behind experience, including variations in the starting age and duration, was not examined in detail. These factors may moderate the intensity of the LBE’s effects. Finally, future research could include moderating variables such as self-esteem and psychological resilience, to provide a more comprehensive understanding of the mechanisms at play, further elucidating the complex relationship between the Left-behind experience and psychological adaptation.

## Conclusion

This study demonstrates that, compared to non-left-behind college students, those with left-behind experiences report significantly lower levels of perceived social support and subjective well-being. Moreover, the reduction in perceived social support mediates the negative effects of the LBE on subjective well-being. These findings emphasize the essential role of developing strong social support systems to mitigate the adverse psychological consequences associated with the left-behind psychological resources to shape long-term well-being, providing valuable insights for promoting the mental health of left-behind individuals.

## Supplemental Information

10.7717/peerj.20567/supp-1 Supplemental Information 1 Data.

10.7717/peerj.20567/supp-2 Supplemental Information 2 Codebook.

## Additional Information and Declarations

### Data Availability

The following information was supplied regarding data availability:

The raw data is available in the Supplemental File.
