# Supplementary material for: Perceived social support as a mediator between left-behind experience and subjective well-being in Chinese college students
Source: PeerJ. 2026 Jan 9;14:e20567. doi: 10.7717/peerj.20567 (PMC12794631; doi:10.7717/peerj.20567)
Supplement: Supplemental Information 2 [file peerj-14-20567-s002.docx]

Supplemental File

Sex： 1—Male；2——Female

Grade;：1——Freshman Year；2——Sophomore year；3——Junior year；4——Senior year

From：1——Town；2——Rural area

Ls: 0——Non-Left-behind.; 1——Left-behind

z1-z12: Score for each question of the Perceived Social Support Scale

x1-x18: Score for each question of Subjective Well-being Scale
